# Can Clustal-style progressive pairwise alignment of multiple sequences be used in RNA secondary structure prediction?

**DOI:** 10.1186/1471-2105-8-190

**Published:** 2007-06-08

**Authors:** Amelia B Bellamy-Royds, Marcel Turcotte

**Affiliations:** 1 School of Information Technology and Engineering, University of Ottawa, Ottawa, Ontario, Canada

## Abstract

**Background:**

In ribonucleic acid (RNA) molecules whose function depends on their final, folded three-dimensional shape (such as those in ribosomes or spliceosome complexes), the secondary structure, defined by the set of internal basepair interactions, is more consistently conserved than the primary structure, defined by the sequence of nucleotides.

**Results:**

The research presented here investigates the possibility of applying a progressive, pairwise approach to the alignment of multiple RNA sequences by simultaneously predicting an energy-optimized consensus secondary structure. We take an existing algorithm for finding the secondary structure common to two RNA sequences, Dynalign, and alter it to align profiles of multiple sequences. We then explore the relative successes of different approaches to designing the tree that will guide progressive alignments of sequence profiles to create a multiple alignment and prediction of conserved structure.

**Conclusion:**

We have found that applying a progressive, pairwise approach to the alignment of multiple ribonucleic acid sequences produces highly reliable predictions of conserved basepairs, and we have shown how these predictions can be used as constraints to improve the results of a single-sequence structure prediction algorithm. However, we have also discovered that the amount of detail included in a consensus structure prediction is highly dependent on the order in which sequences are added to the alignment (the guide tree), and that if a consensus structure does not have sufficient detail, it is less likely to provide useful constraints for the single-sequence method.

## 1 Background

The research presented here investigates the possibility of applying a progressive, pairwise approach to the alignment of multiple ribonucleic acid (RNA) sequences, in which the property being aligned is not the primary structure defined by the identity of the nucleotides, but the secondary structure created from base pair interactions. In RNA molecules whose function depends on their final, folded three-dimensional shape (such as those in ribosomes or spliceosome complexes), the secondary structure is more consistently conserved than the sequence.

Although there exist dynamic-programming methods for predicting the secondary structure of a single sequence, the quality of the prediction can be significantly improved by using an alignment of multiple related sequences; unfortunately, it is difficult to accurately align the sequences without knowing their structures. A theoretical algorithm was proposed by Sankoff more than twenty years ago [1] to solve the alignment and structure prediction problems simultaneously. Although a variety of programs (e.g. Foldalign [2], PMcomp/PMmulti [3]) are based on restricted versions of the Sankoff algorithm, it is so computationally complex in time and memory requirements that it has never been implemented in full.

Similar limitations exist when aligning multiple sequences using dynamic programming approaches. The exact algorithm is unreasonably slow for very long sequences (e.g. genomes), or for sets of many sequences. Hence, heuristic methods have been developed. Clustal [4] is a widely used multiple sequence alignment program, which usually produces results that are reliable enough that the extra resources required by the full algorithm are rarely justified. The heuristic used by Clustal is to approach the alignment of the entire set of sequences through a progressive series of pairwise alignments, creating profiles of partial sets of sequences which are then fixed in relative alignment when that profile is aligned to additional sequences. The quality of the multiple sequence alignment produced by the Clustal series of programs has been improved by a number of adjustments as the program has been updated over the years. One essential heuristic is that the order in which sequences are aligned reflects an estimated phylogenetic tree, such that the most closely related sequences are aligned first, while more diverse sequences – which have a greater possibility of being aligned incorrectly – are added later in the process. The tree structure that is used to guide the alignment is calculated based on the relative scores of alignments of all possible pairs of individual sequences.

Here, we take a constrained, pairwise implementation, Dynalign [5], and alter it to align profiles of multiple sequences, and then explore the relative successes of different approaches to designing the tree that will guide progressive pairwise alignments of sequence profiles to create a multiple sequence alignment and a consensus prediction of a conserved secondary structure.

### 1.1 Background: RNA alignment and secondary structure prediction

The 'traditional' methods to determine the secondary structure of RNA involve a combination of biochemical analysis of folded molecules and comparative sequence analysis of a gene family with known phylogeny. By analyzing the covariance between particular nucleotides across a set of sequences, probable basepairs can be detected by the fact that mutations in the primary sequence will maintain the secondary-structure pairing pattern. The combination of these two approaches, applied to the most intensively studied classes of structural RNA molecules (ribosomal RNA and transfer RNA), have resulted in detailed structural models which have been supported by more recent three-dimensional X-ray crystallography analyses [6]. However, both approaches are extremely time-consuming, and covariance analyses require large numbers of sequences, including closely related molecules which can be the basis of reliable alignments of the primary sequences. As more and more classes of functional RNA molecules are discovered (see the review by Storz [7] for examples), reliable computational methods for detecting conserved RNA secondary structure would increase the ability to connect sequence data to structure and function.

There are many different computational approaches currently being used to attempt to predict secondary structure of single RNA molecules, or to detect conserved structures or structural motifs in multiple sequences. The approach described here focuses on the global alignment and structure prediction of a set of related sequences, using the nearest-neighbour energy model to assess the probable stability of different potential structures.

The nearest-neighbour energy model is a rule-based approach which attempts to explain empirically-measured thermodynamic changes in the stability of different RNA molecules, using a sum of energy terms representing the theoretical creation of the complete structure from smaller structures by adding nucleotides (possibly base-paired) at either end of the sub-structure. Each energy term is only dependent on the identity of the bases added and those at the ends of the sub-structure (the 'nearest neighbours'). The model is therefore ideally suited for a dynamic programming calculation, since the optimal energy conformation for subsequences of a certain length can be calculated by extending previously calculated shorter sub-structures; the process is repeated starting with small, trivial cases (hairpin loops), until a structure for the entire sequence is predicted [8].

As mentioned, the energy terms are derived from empirical thermodynamic measurements (changes in the 'melting temperature' between similar molecules). In order to explain as much of the variance of the empirical data as possible, the model uses a complex set of rules and terms, accounting for diverse possible interactions between adjacent bases which could increase or decrease the stability of the structure. However, in order to maintain the computational simplicity of the dynamic programming method, tertiary (three-dimensional) interactions, which may significantly affect stability, are not considered. Energy minimization methods also do not consider kinetic effects which may favour a locally stable conformation over a global optimum.

For these and related reasons, many single-sequence secondary structure prediction programs based on the nearest-neighbour model, such as the commonly used mfold [9], currently predict multiple potential structures if they have similar calculated stability energies, and also recalculate the energies for the potential structures using a more complex energy model. However, even with these adjustments, the nearest-neighbour model is still only a rough approximation of the true change in free energy between the unfolded and folded structures, and some structures are very poorly predicted by this method alone.

The predictions from the nearest neighbour energy model can be improved by incorporating covariance information from multiple related sequences, to distinguish conserved basepairs from potential interactions which are not part of the biologically significant structure. The program RNAalifold [10], which applies a nearest-neighbour model to a precalculated sequence alignment, can significantly improve structure predictions relative to single-sequence methods, but only if the input alignment is reliable [11]. This is an important caveat, since sequence alignment methods developed for amino acid sequences or protein-encoding nucleic acids assume patterns of sequence conservation different from the structural RNA molecules. In order to generate a reliable alignment of RNA sequences without prior structural knowledge, the primary sequences must be near-identical; however, if the sequences are too similar, then the alignment does not add significant information, and the structure predicted for the profile may be no better than predictions for the individual sequences.

The program Dynalign, developed by Mathews and Turner [5], attempts to circumvent this problem by combining alignment and structure prediction into one dynamic-programming optimization. Their program is essentially a restricted implementation of an algorithm originally proposed by Sankoff [1]. Sankoff's general algorithm (never implemented) describes how a set of nucleic acid sequences with unknown conserved secondary structure could be aligned, and the conserved structure predicted, by calculating the most stable conserved structure for all possible alignments of one subsequence from each sequence in the set, by extending structures for alignments of the same or shorter subsequences. Since the number of subsequences of a sequence is proportional to the square of its length, the time required for a complete implementation of the algorithm would be proportional to the product of the squares of the lengths of all sequences in the set (O
 MathType@MTEF@5@5@+=feaafiart1ev1aaatCvAUfKttLearuWrP9MDH5MBPbIqV92AaeXatLxBI9gBamrtHrhAL1wy0L2yHvtyaeHbnfgDOvwBHrxAJfwnaebbnrfifHhDYfgasaacH8akY=wiFfYdH8Gipec8Eeeu0xXdbba9frFj0=OqFfea0dXdd9vqai=hGuQ8kuc9pgc9s8qqaq=dirpe0xb9q8qiLsFr0=vr0=vr0dc8meaabaqaciaacaGaaeqabaWaaeGaeaaakeaaimaacqWFoe=taaa@383D@(*n*^2*k*^), for *k *sequences of length *n*); energy values for all the possible sub-alignments would have to be maintained in order to calculate longer alignments, so the memory requirements would increase in the same proportions.

Dynalign limits the computational complexity of the algorithm by only aligning two sequences, and by restricting the change in index between aligned nucleotides in the two sequences to be no greater than a chosen maximum separation distance: for example, with maximum separation *m*, the nucleotide at index *i *of the first sequence could only be aligned with nucleotides at indices *i *± *m *of the second sequence. This constraint significantly reduces the number of different subsequences compared, limiting the overall time and memory complexity to O
 MathType@MTEF@5@5@+=feaafiart1ev1aaatCvAUfKttLearuWrP9MDH5MBPbIqV92AaeXatLxBI9gBamrtHrhAL1wy0L2yHvtyaeHbnfgDOvwBHrxAJfwnaebbnrfifHhDYfgasaacH8akY=wiFfYdH8Gipec8Eeeu0xXdbba9frFj0=OqFfea0dXdd9vqai=hGuQ8kuc9pgc9s8qqaq=dirpe0xb9q8qiLsFr0=vr0=vr0dc8meaabaqaciaacaGaaeqabaWaaeGaeaaakeaaimaacqWFoe=taaa@383D@(*n*^2^*m*^2^); however, it does mean that Dynalign is only appropriate for global alignments (not local alignments or motif detection).

In order to model potential deletion or insertion mutations between the two sequences, Dynalign allows for the addition of gaps to the alignment. However, to avoid having the program insert excessive gaps while optimizing incorrect basepairs, an additional term is included in the energy calculations to penalize added gaps. Unlike the other terms, there is no empirically-derived value for the gap penalty, so Mathews and Turner explored the effect of a series of values. Their results were not conclusive; they found that the optimum value depended on the data set being examined, although a penalty below optimum resulted in poorer predictions then a penalty the same distance above optimum [5].

In many cases, Dynalign calculations can avoid incorrect predictions made by mfold, but because it only uses two sequences, the available covariance information is limited. For this reason, researchers in our lab have previously implemented an extended version of Dynalign (X-Dynalign), which finds the common structure and alignment of three input sequences [12]. We found that this approach increased the reliability of the predictions (it was less likely to predict biologically non-meaningful basepairs), and overall improved the worst case predictions. The X-Dynalign experiments also showed that lower-than-optimum gap penalties were likely to cause poor results, while at higher penalties results were more consistent. The computational complexity of the extended program increases to O
 MathType@MTEF@5@5@+=feaafiart1ev1aaatCvAUfKttLearuWrP9MDH5MBPbIqV92AaeXatLxBI9gBamrtHrhAL1wy0L2yHvtyaeHbnfgDOvwBHrxAJfwnaebbnrfifHhDYfgasaacH8akY=wiFfYdH8Gipec8Eeeu0xXdbba9frFj0=OqFfea0dXdd9vqai=hGuQ8kuc9pgc9s8qqaq=dirpe0xb9q8qiLsFr0=vr0=vr0dc8meaabaqaciaacaGaaeqabaWaaeGaeaaakeaaimaacqWFoe=taaa@383D@(*n*^3^*m*^6^) in time and O
 MathType@MTEF@5@5@+=feaafiart1ev1aaatCvAUfKttLearuWrP9MDH5MBPbIqV92AaeXatLxBI9gBamrtHrhAL1wy0L2yHvtyaeHbnfgDOvwBHrxAJfwnaebbnrfifHhDYfgasaacH8akY=wiFfYdH8Gipec8Eeeu0xXdbba9frFj0=OqFfea0dXdd9vqai=hGuQ8kuc9pgc9s8qqaq=dirpe0xb9q8qiLsFr0=vr0=vr0dc8meaabaqaciaacaGaaeqabaWaaeGaeaaakeaaimaacqWFoe=taaa@383D@(*n*^2^*m*^4^) in memory; even with the fairly restrictive maximum separation value of 5, calculations for 5S rRNA sequences (length approximately 120 nucleotides) required many days and gigabytes of memory to complete. Any extension to increase the number of sequences beyond three would be computationally infeasible with current computing technology.

Other approaches have been used to modify Sankoff's algorithm into an implementable program. For example, the program PMmulti [3], which avoids simultaneously solving the folding and alignment problems, by first computing base pairing probability matrices for each sequence using the McCaskill approach. It then extracts a common secondary structure and alignment using a dynamic programming approach akin to Nussinov's weighted circular matching algorithm.

### 1.2 Progressive pairwise alignment of RNA sequences

In order to search for a common structure in a set of more than three related RNA sequences, we have adopted the progressive pairwise alignment approach exemplified by the Clustal programs for primary sequence multiple alignments. The concept is to find a common structure between a pair of sequences, using the Dynalign algorithm, and then use the resulting alignment of those sequences as a fixed profile in order to find a common structure between them and an additional sequence or profile. The approach is repeated, aligning sequences and profiles in an order defined by the nodes of a binary tree with the individual sequences at the leaves, until the entire data set has been aligned and a consensus structure predicted for the final alignment. For a data set of *k *sequences, this will require *k *- 1 iterations of the program, making the overall time complexity of the program O
 MathType@MTEF@5@5@+=feaafiart1ev1aaatCvAUfKttLearuWrP9MDH5MBPbIqV92AaeXatLxBI9gBamrtHrhAL1wy0L2yHvtyaeHbnfgDOvwBHrxAJfwnaebbnrfifHhDYfgasaacH8akY=wiFfYdH8Gipec8Eeeu0xXdbba9frFj0=OqFfea0dXdd9vqai=hGuQ8kuc9pgc9s8qqaq=dirpe0xb9q8qiLsFr0=vr0=vr0dc8meaabaqaciaacaGaaeqabaWaaeGaeaaakeaaimaacqWFoe=taaa@383D@(*kn*^2^*m*^2^); the memory requirements remain O
 MathType@MTEF@5@5@+=feaafiart1ev1aaatCvAUfKttLearuWrP9MDH5MBPbIqV92AaeXatLxBI9gBamrtHrhAL1wy0L2yHvtyaeHbnfgDOvwBHrxAJfwnaebbnrfifHhDYfgasaacH8akY=wiFfYdH8Gipec8Eeeu0xXdbba9frFj0=OqFfea0dXdd9vqai=hGuQ8kuc9pgc9s8qqaq=dirpe0xb9q8qiLsFr0=vr0=vr0dc8meaabaqaciaacaGaaeqabaWaaeGaeaaakeaaimaacqWFoe=taaa@383D@(*n*^2^*m*^2^).

In implementing and testing this approach, we have explored the effect of the order in which sequences are added to the alignment on the quality of the eventual predictions. For randomized sequence orders, we consider the effect of the topology of the guide tree: whether it has a balanced or linear structure. For an approximate phylogenetic order, we consider two ways of constructing a guide tree: by using the phylogeny predicted by Clustal W based purely on primary sequence, and by using a neighbour-joining algorithm to create a tree based on the energy scores of all possible pairwise Dynalign alignments. Throughout, we have also investigated the optimization of the Dynalign gap penalty term.

**Figure 1:**
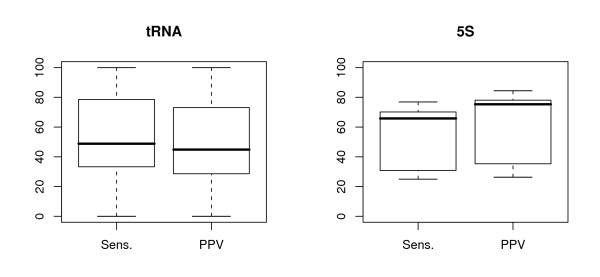
Boxplots showing the range of sensitivity and positive predictive value statistics for mfold optimum predictions on the two test data sets.

The programs have been run on two different data sets of twelve sequences each, one of transfer RNA (tRNA) sequences from diverse organisms, and the other using the 5S RNA molecule from bacterial large ribosomal subunits (5S rRNA), see Section 4.1 for details. All of the sequences have reliable reference structures derived from comparative sequence analysis, but many of the structures are poorly predicted by single-sequence methods, see Figure 1. We compare the single-sequence folding program mfold, the two-sequence Dynalign, and our various experimental approaches to creating a consensus alignment, for their ability to predict these reference structures.

Because the purpose of the method is to find a strict consensus structure that exists in all sequences in the data set, it is not expected to detect any structural details unique to particular sequences: it will not predict a particular basepair for the alignment as a whole unless it is possible in every sequence at that point. For these reasons, the predicted structure for any one sequence may not be as complete as a prediction made with a single-sequence method. Furthermore, the Dynalign energy model only considers canonical base pairing interactions (the Watson-Crick C:G and A:U pairs, plus the 'wobble' G:U pair); other pairing interactions detected through comparative sequence analysis or biochemical means will not be predicted. Finally, like most other dynamic-programming structure prediction algorithms, it will not predict any pseudoknot structures: structures in which a nucleotide contained within a segment bound by a base-pair itself pairs to a nucleotide outside that segment. With all that said, the hope is that the predictions will be highly reliable, as there is unlikely to be a biologically irrelevant nearest-neighbour optimum structure common to the entire data set.

### 1.3 Assessing results

In order to compare the accuracy of the various methods in predicting the reference structures, some quantitative measures of similarity are required. The *Sensitivity *of a prediction is defined as the percentage of basepairs in the reference structure which were predicted, while the *Positive Predictive Value *(PPV) is the percentage of predicted basepairs that also exist in the reference structure. A prediction with low sensitivity therefore does not include all the detail of the true structure, while a prediction with low PPV includes a number of inaccurate predictions (PPV is undefined if a method does not produce any predicted basepairs at all). We use a strict definition of a correctly predicted basepair – the exact same nucleotides must be paired in both structures – and consider all incorrect predictions equally.

A third statistic, used to combine these two measures into an overall assessment of the quality of prediction, is the *Matthew's Correlation Coefficient *(MCC). For the calculations, we used the approximation proposed by Gorodkin *et al.*, where MCC is calculated as the square root of the product of the sensitivity and PPV statistics [2].

## 2 Results and discussion

### 2.1 Reference alignment and structures

The ID codes, source species, primary sequences and reference structures for all twelve tRNA sequences are given in Table 1. The tRNA sequences include those with and without large loops or additional helices in the variable region of the standard 'cloverleaf' structure; the total length of the sequences ranges from 71 to 92 nucleotides. The pairwise primary sequence identity (based on the reference alignment) ranges from 26% to 68%; the average is 43.5%.

**Table 1:** The tRNA sequences in the test data set. Reference alignment showing the reference structures and the ideal consensus structure (bottom). Quotes indicate non-canonical basepairs. The number of nucleotides and of basepairs is given for each sequence and structure.

**ID & Species**	**Sequence & Structure**	**Length**	**ID**
**RD0260**	GCGACCGGGGCUGGCUU-GGUA-AUGGUACUCCCCUGUCACGGGAGAG---------------AAUGUGGGUUCAAAUCCCAUCGGUCGCGCCA	77	**RD0260**
Phage T5 (Virus)	(((((((..((((....-....-.)))).(((((.......)))))..---------------...(((((.......))))))))))))....	21	
**RD0500**	GCCCGGGUGGUGCAGU--GGCCCAGCAUACGACCCUGUCACGGUCGUG---------------A-CGCGGGUUCAAAUCCCGCCUCGGGCGCCA	76	**RD0500**
*Haloferax volcanii *(Archæa)	(((((((..((((...--......)))).(((((.......)))))..---------------.-.(((((.......))))))))))))....	21	
**RD1140**	GGCCCCAUAGCGAAGUU-GGUU-AUCGCGCCUCCCUGUCACGGAGGAG---------------AUCACGGGUUCGAGUCCCGUUGGGGUCGCCA	77	**RD1140**
*Mycoplasma capricolum *(Eubacteria)	(((((((..((((....-....-.)))).(((((.......)))))..---------------...(((((.......))))))))))))....	21	
**RD2640**	GGGAUUGUAGUUCAAUU-GGUC-AGAGCACCGCCCUGUCAAGGCGGAA---------------GAUGCGGGUUCGAGCCCCGUCAGUCCCGCCA	77	**RD2640**
*Hordeum vulgare *(Plant chloroplast)	(((((((..((((....-....-.)))).(((((.......)))))..---------------...(((((.......))))))))))))....	21	
**RD4800**	AAAAAAUUAGUUUAAU--CA---AAAACCUUAGUAUGUCAAACUAAAA---------------A-AAUUAGAUCAU--CUAAUAUUUUUUACCA	71	**RD4800**
*Aedes albopictus *(Animal mitochondria)	(((((((..((((...--..---.)))).(((((.......)))))..---------------.-.(((((.....--))))))))))))....	21	
**RE2140**	GCCCCCAUCGUCUAGA--GGCCUAGGACACCUCCCUUUCACGGAGGCG---------------A-CAGGGAUUCGAAUUCCCUUGGGGGUACCA	76	**RE2140**
*Synechocystis *sp. (Eubacteria)	(((((((..((((...--......)))).(((((.......)))))..---------------.-.(((((.......))))))))))))....	21	
**RE6781**	UCCGUCGUAGUCUAGGUGGUUA--GGAUACUCGGCUCUCACCCGAGAG---------------A-CCCGGGUUCGAGUCCCGGCGACGGAACCA	76	**RE6781**
*Hordeum vulgare *(Plant chloroplast)	(((((((..((((.........--)))).(((((.......)))))..---------------.-.(((((.......))))))))))))....	21	
**RF6320**	GUCGCAAUGGUGUAGUUGGGA---GCAUGACAGACUGAAGAUCUGUUG---------------GUCAUCGGUUCGAUCCCGGUUUGUGACACCA	76	**RF6320**
*Schizosaccharomyces pombe *(Fungi cytoplasm)	(((((((..((((........---)))).(((((.......)))))..---------------...(((((.......))))))))))))....	21	
**RL0503**	GCGGGGGUGGCUGAGCCAGGCCAAAAGCGGCGGACUUAAGAUCCGCU-CCC--GUAG---GGGUUCGCGAGUUCGAAUCUCGUCCCCCGCACCA	88	**RL0503**
*Haloferax volcanii *(Archæa)	(((((((..((('...........'))).(((((.......))))).-(((--....---)))...(((((.......))))))))))))....	24	
**RL1141**	CCCCAAGUGGCGGAAUA-GGDA-GACGCAUUGGACUUAAAAUCCAAC-GGGC-UUAAU-AUCCUGUGCCGGUUCAAGUCCGGCCUUGGGGACCA	89	**RL1141**
*Mycoplasma capricolum *(Eubacteria)	(((((((..(((..... -.... -..))).(((((.......))))).-.... -.....-.......(((((.......))))))))))))....	20	
**RS0380**	GCCGAGGUAGCAUAGCUUGGCC-AAUGCGGUUGCUUCGAGAGCAACG-UUCC-ACAC--GGACU-CAGGAGUUCAAAUCUCCUCCUCGGCGCCA	88	**RS0380**
*Halobacterium cutirubrum *(Archæa)	(((((((..((((.........-.)))).(((((.......))))).-'(((-....--)))'.-.(((((.......))))))))))))....	25	
**RS1141**	GGAAGAUUACCCAAGUCCGGCUGAAGGGAUCGGUCUUGAAAACCGAGAGUCGGGGAAACCGAG--CGGGGGUUCGAAUCCCUCAUCUUCCGCCA	92	**RS1141**
*Mycoplasma capricolum *(Eubacteria)	(((((((..((('...........'))).(((((.......)))))..'((((.....))))'--.(((((.......))))))))))))....	26	

**Consensus structure**	(((((((..(((.............))).(((((.......))))).................... (((((.......))))))))))))....	20	**Consensus**

The reference sequences and structure alignment for the 5S rRNA data set are given in Table 2. The pairwise primary sequence identity ranges from 55% to 100% (the 5S rRNAs from *Arthrobacter oxydans*, X08000, and *A. globiformis*, X08002, have the same primary sequence); the average pairwise identity is 67%.

**Table 2:** The bacterial 5S rRNA sequences in the test data set. Reference alignment showing the reference structures and the ideal consensus structure (bottom). Quotes indicate non-canonical basepairs. The number of nucleotides and of basepairs is given for each sequence and structure.

**ID & Species**	**Sequence & Structure**	**Length**
**AJ131594**	-UGCC-UGAUGACCAUAGCAAGUUGGUACCACUCCUUCCCAUCCCGAACAGGACAGUGAAA-CGACUUUGCGCCGAUGAUAGUGCGG--GUU---CCCGUGUGAAAGUAGGUCAUCGUCAGGCNN---	117
*Comamonas acidovorans*	-'(((-((((((..... ((((((((....(((((((.............))))..)))...-)))))).)).(('((''..((((((--...---.))))))..''))'))...)))))))))'.---	38
**AJ251080**	---CC-UAGUGGUGAUAGCGGAGGGGAAACACCCGUUCCCAUCCCGAACACGGAAGUUAAG-CCCUCCAGCGCCGAUGGUAGUUGGGGCCAGC-GCCCCUGCAAGAGUAGGUCGCUGCUAGG----C-	117
*Bacillus stearothermophilus*	---((-((((((.....((((((((....'((((((.............))))..))'...-)))))).)).(('((''..(('(((((....-)))))'))..''))'))...))))))))----.-	38
**K02682**	-NGUC-UGGCGGCCAUAGCGUGGGGGAAACGCCCGGUCCCAUCCCGAACCCGGAAGCUAAG-CCCCAUAGCGCCGAUGGUACUGCAACCGGGA-GGUUGUGGGAGAGUAGGUCGCCGCCGGAC---A-	120
*Micrococcus luteus*	-.(((-((((((.....((((((((....'((((((.............))))..))'...-)))))).)).(('((''.. ((((((((....-))))))))..''))'))...)))))))))---.-	39
**M10816**	-NNCC-UAGUGACAAUAGCGGAGAGGAAACACCCGUUCCCAUCCCGAACACGGAAGUUAAG-CUCUCCAGCGCCGAUGGUAGUUGGGGCCAGC-GCCCCUGCAAGAGUAGGUCGUUGCUAGG----C-	119
*Bacillus stearothermophilus*	-..((-((((((..... ((((((((....'((((((.............))))..))'...-)))))).)).(('((''.. (('(((((....-)))))'))..''))'))...))))))))----.-	38
**M16532**	-NCUC-GGACCACCAUACCGGGGGGGAAACACCCGGUCCCAUUCCGAACCCGGAAGUUAAG-CCCCCCAGGGCCGAUGAUAGCCUCGC-CCCGA-GCGAGGUGAAAGUAGGUCGUGGUCCGGGCAC--	121
*Thermus *sp.	-'(((-((((((.....((((((((....'((((((.............))))..))'...-)))))).)).(('((''..(((((((-.....-)))))))..''))'))...)))))))))'..--	39
**M25591**	---CC-UAGUGGUGAUAGCGGAGGGGAAACACCCGUUCCCAUCCCGAACACGGAAGUUAAG-CCCUCCAGCGCCGAUGGUAGUUGGGGCCAGC-GCCCCUGCAAGAGUAGGCCGCUGCUAGG----C-	117
*Bacillus stearothermophilus*	---((-((((((.....((((((((....'((((((.............))))..))'...-)))))).)).(('((''..(('(((((....-)))))'))..''))'))...))))))))----.-	38
**V00336**	-UGCC-UGGCGGCCGUAGCGCGGUGGUCCCACCUGACCCCAUGCCGAACUCAGAAGUGAAA-CGCCGUAGCGCCGAUGGUAGUGUGGGGUCU--CCCCAUGCGAGAGUAGGGAACUGCCAGGCA--U-	120
*Escherichia coli*	-((((-((((((.....((((((((....(((((((.............))))..)))...-)))))).)).(('((''..((((((((...--))))))))..''))'))...))))))))))--.-	40
**X02024**	-NNUU-UGGUGGCGAUAGCGAAGAGGUCACACCCGUUCCCAUACCGAACACGGAAGUUAAG-CUCUUCAGCGCCGAUGGUAGUUGGGGUGUUA-GCCCCUGCAAGAGUAGGACGUUGCCAGG----C-	119
*Bacillus pasteurii*	-..((-((((((.....((((((((....'((((((.............))))..))'...-)))))).)).(('((''..(('(((((....-)))))'))..''))'))...))))))))----.-	38
**X02627**	CGACC-UGGUGGUCAUCGCGGGGCGGCUGCACCCGUUCCCUUUCCGAACACGGCCGUGAAA-CGCCCCAGCGCCAAUGGUACUUCGUC-UCAA--GACGCGGGAGAGUAGGUCGCUGCCAGGUC---U	120
*Agrobacterium tumefaciens*	.((((-((((((.....((((((((....(((((((.............))))..)))...-)))))).)).(('((''..(('((((-....--))))'))..'')) '))...))))))))))---.	39
**X04585**	-CGUU-UGGUGGUCAUAGCGUUGGCUAAACACCCGAUCCCAUCCCGAACUCGGCCGUUAAGGGCCAACA-CGCCGAUGGUACUGCGUC-UCAA--GACGUGGGAGAGUAGGUCACCGCCAAACCN---	119
*Rhodobacter capsulatus*	-'(((-((((((.....(.((((((....'((((((.............))))..))'....)))))).-).(('((''..(((((((-....--)))))))..''))'))...)))))))))'.---	38
**X08000**	-NAUUACGGCGGUCAUAGCGUGGGGGAAACGCCCGGUCCCAUUCCGAACCCGGAAGCUAAG-ACCCACAGCGCCGAUGGUACUGCACCCGGGA-GGGUGUGGGAGAGUAGGUCACCGCCGGACAC---	122
*Arthrobacter oxidans*	-.'((.((((((.....((((((('....'((((((.............))))..))'...-'))))).)).(('((''..((((((((....-))))))))..''))'))...))))))))'..---	39
**X08002**	-NAUUACGGCGGUCAUAGCGUGGGGGAAACGCCCGGUCCCAUUCCGAACCCGGAAGCUAAG-ACCCACAGCGCCGAUGGUACUGCACCCGGGA-GGGUGUGGGAGAGUAGGUCACCGCCGGACAC---	122
*Arthrobacter globiformis*	-.'((.((((((.....((((((('....'((((((.............))))..))'...-'))))).)).(('((''..((((((((....-))))))))..''))'))...))))))))'..---	39

**Consensus Structure**	...((.((((((.....(.(((((......((((((.............))))..))......)))))..).((.((....((.(((.........))).))....)).))...))))))))......	29

For both data sets, an 'ideal' consensus structure, based on the reference alignment and individual structures, has been created and is also shown in the sequence tables. The consensus only contains basepairs where all sequences have a canonical basepair at that position in the alignment, and therefore represents the best possible prediction one could expect from the progressive Profile-Dynalign method. The tRNA consensus structure contains 20 basepairs, as compared to 20–26 basepairs in the individual structures. The 5S consensus contains 29 basepairs, compared to 38–40 basepairs per structure (of which 33–37 basepairs per structure are canonical). However, because the consensus structures are missing basepairs in some helical regions, it is possible that the calculated stability of these helices will be reduced to the point where they are not predicted at all.

### 2.2 Single sequence folding using mfold

The secondary structure predictions from mfold vary widely in quality for our data sets, but are generally poor: these sequences were originally selected to demonstrate the potential for improvement over single-sequence structure prediction methods. Considering only the structures reported by mfold as the energy-model optimum, the worst prediction was for tRNA sequence RF6320, in which none of the predicted basepairs are present in the reference structure; the best prediction was for tRNA sequence RD1140, in which the reference structure was perfectly predicted. For the 5S rRNA sequences, the worst was for sequence V00336, with an MCC value of 0.256, while the best was sequence M16532, with MCC = 0.801. The overall range in values for each data set is charted in Figure 1. In general, the sensitivity and predictive value are similar for a given structure: when a prediction fails to include reference basepairs, it is usually predicting incorrect pairs instead.

### 2.3 Pairwise alignment and folding

The alignment and structure prediction of all the possible pairs of sequences resulted in 11 predictions for each of 12 sequences, at each of 7 gap penalty levels tested, for each of the two data sets. For the tRNA sequences, the worst prediction (0.240 MCC) was for sequence RS1141 when aligned with RE2140 at gap penalty 0.5 kcal/mol; however, many other pairs resulted in perfect predictions. For the 5S rRNA sequences, the worst prediction was for sequence M25591 when aligned with sequence AJ131594 with zero gap penalty (0.165 MCC); the best prediction was for sequence K02682 when aligned with AJ131594 with gap penalty 4.0 kcal/mol (0.768 MCC, with perfect predictive value).

**Figure 2:**
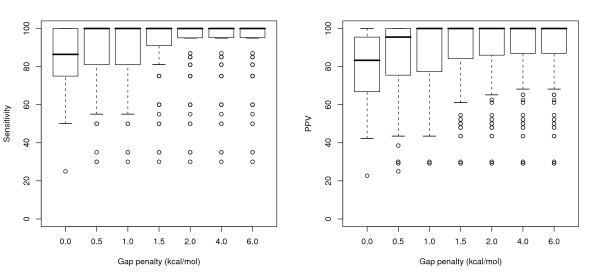
Boxplots showing the range of sensitivity and positive predictive value statistics for all pairwise alignments of the tRNA sequences, at each gap penalty value tested.

**Figure 3:**
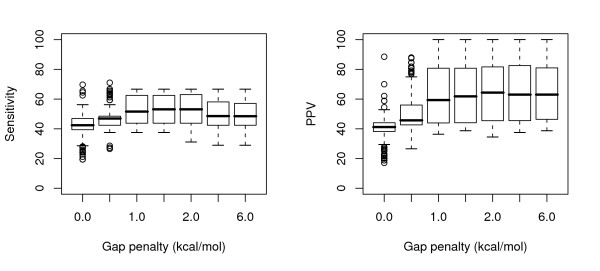
Boxplots showing the range of sensitivity and positive predictive value statistics for all pairwise alignments of the 5S rRNA sequences, at each gap penalty value tested.

The overall range in both sensitivity and PPV values at each gap penalty level is shown in Figure 2 for tRNAs and Figure 3 for 5S rRNA sequences. As can be seen, for the tRNA sequences, the results are fairly consistent for gap penalties ranging from 0.5 kcal/mol to 6.0 kcal/mol (the highest value tested); this loosely correlates with results from the original paper [5], despite the use of different parameters (increased maximum separation and inserts allowed in helices). The majority of predictions in these trials had perfect PPV and near-perfect sensitivity, but there nonetheless remains a long 'tail' of poor results at each penalty level.

The predictions for the 5S rRNAs show a stronger relationship to the change in gap penalty, with a clear optimum in sensitivity for penalty values between 1 and 2 kcal/mol, while the predictive value was fairly constant with a gap penalty greater than 1 kcal/mol. With the same parameters, but a different set of 5S rRNA sequences, Mathews and Turner had found that the results peaked at a penalty of 0.4 kcal/mol; for this set, a penalty of 0.5 kcal/mol (the nearest value tested) produced noticeably less than optimal predictions.

### 2.4 Increasing the number of sequences

**Figure 4:**
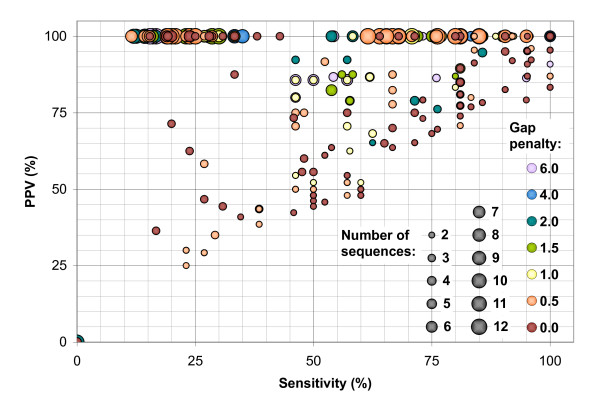
Sensitivity versus positive predictive value (PPV) for all predicted tRNA structures from the pairwise alignments and all stages of the randomized progressive alignments, with the gap penalty used and the number of sequences in the alignment indicated by the colour and size of the points, respectively.

**Figure 5:**
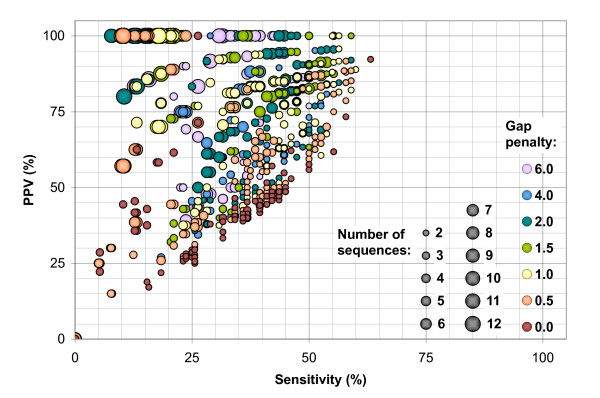
Sensitivity versus positive predictive value (PPV) for all predicted 5S rRNA structures from the pairwise alignments and all stages of the randomized progressive alignments, with the gap penalty used and the number of sequences in the alignment indicated by the colour and size of the points, respectively.

As the number of sequences in an alignment is increased by the progressive pairwise approach, a number of trends in the quality of the resulting structural predictions become apparent. Figure 4 compares the sensitivity and PPV statistics for the predictions for each tRNA sequence, relative to the number of sequences in an alignment and the gap penalty used, for all the pairwise results and all the randomized tree structures sampled. Figure 5 shows the same data for the 5S rRNA sequences.

As expected, the sensitivity of the predictions is reduced and the predictive value increased as the number of sequences used increases. However, the loss in sensitivity can be quite drastic, and frequently results in no predicted structure at all for alignments of more than 6 or 7 sequences. Nonetheless, when a structure is predicted for alignments of this size, the basepairs are nearly always part of the reference conserved structures.

The drop in prediction quality at low gap penalty values is even more pronounced. Zero gap penalty always resulted in a lack of predicted structure: the alignments produced generally have so many gaps added that the difference in length between partial alignments and new sequences is greater than the maximum separation parameter (results not shown). For the tRNA data set, all values above this have similar results, except for an additional drop in sensitivity at the highest gap penalty tested (6 kcal/mol). For the 5S rRNA sequences, the results for a gap penalty of 0.5 kcal/mol were also poor, but above this were fairly constant up until 6 kcal/mol, at which point sensitivity was often higher, but PPV sometimes lower.

It should be emphasized that, due to the complexity of the programs, only six randomized trees (three balanced and three random) were run for each data set at each gap penalty level. However, there are hundreds of millions of possible binary tree topologies with twelve distinct leaf-nodes, so that differences between gap penalty levels may be due to sampling error.

### 2.5 Optimizing the tree structure

**Figure 6:**
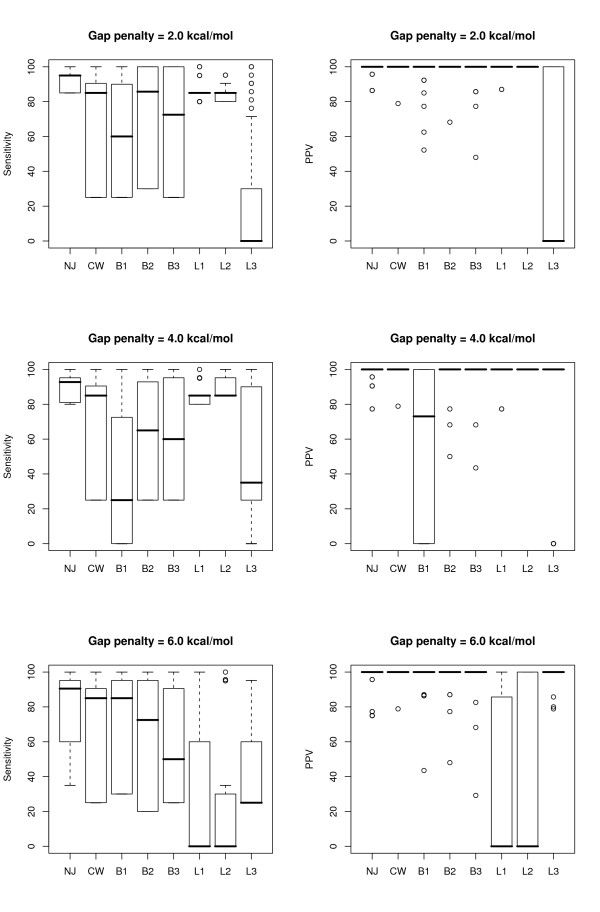
Boxplots showing the range in sensitivity and positive predictive value (PPV) for the twelve predicted tRNA structures from the final alignment and consensus predictions of all runs with gap penalty ≥ 2.0 kcal/mol, defined by the algorithm used to build the guide tree. NJ is neighbor-joining, CW is Clustal W, B1, B2 and B3 represent the 3 runs with balanced guide trees, while L1, L2 and L3 represent the 3 runs with linear guide trees. Undefined PPV values are plotted as zero.

**Figure 7:**
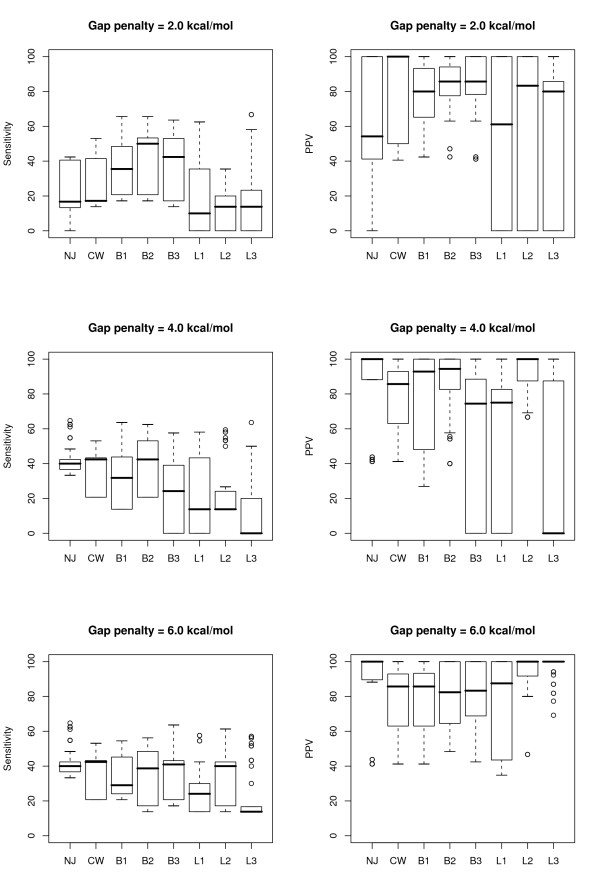
Boxplots showing the range in sensitivity and positive predictive value (PPV) for the twelve predicted 5S rRNA structures from the final alignment and consensus predictions of all runs with gap penalty ≥ 2.0 kcal/mol, defined by the algorithm used to build the guide tree. NJ is neighbor-joining, CW is Clustal W, B1, B2 and B3 represent the 3 runs with balanced guide trees, while L1, L2 and L3 represent the 3 runs with linear guide trees. Undefined PPV values are plotted as zero.

For the final twelve-sequence alignments based on a randomized input order, the 'one-at-a-time' linear tree topology seemed to frequently result in better alignments (and therefore more sensitive consensus structures) for the tRNA data set than when a balanced tree was used as a guide. For the 5S rRNA sequences the opposite seemed to be true – based on the limited number of random trees used – but the difference was not as great. Nonetheless, both types of randomized trees frequently resulted in alignments from which the algorithm could find no thermodynamically stable consensus structure. The range in prediction statistics for the final consensus structures created with the various different guide-tree algorithms are shown in Figure 6 for tRNA molecules, and Figure 7, for 5S rRNAs, for select gap penalty values.

The guide trees produced by applying the neighbour-joining algorithm to the pairwise Dynalign energy values ensured results that were as good as or better than the best results from the randomized trees, for gap penalty values greater than or equal to a data set-dependent minimum. For the tRNA data set, this was 1 kcal/mol, but for the 5S rRNA set, the neighbour-joining trees did not predict any consensus structure for gap penalty values less than 4 kcal/mol; nonetheless, for gap penalties of 4 or 6 kcal/mol, this approach resulted in a prediction of 11 consensus base-pairs, more than were predicted by any of the randomized trees.

As with the randomized trees, once a full alignment of all twelve sequences was reached, all the predicted pairs for the tRNA consensus reflected reference basepairs, while at most one pair was misaligned in some of the 5S sequences.

Since the pairwise alignment scores used to create the neighbour-joining tree are time-consuming to generate, we also ran the progressive alignment on a set of pairwise results calculated with a restricted maximum separation parameter. This appeared to be a relatively successful way to speed up the creation of the guide tree. Although the trees sometimes varied slightly from those created from the full alignment scores, the final consensus predictions were the same for the 5S sets, and had at most one missing basepair for the tRNA predictions (results not shown).

**Figure 8:**
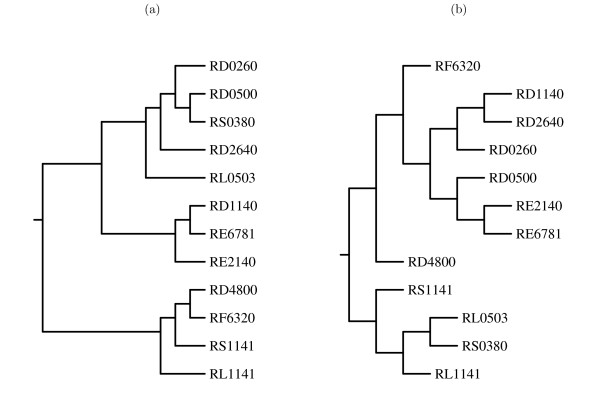
The phylogeny predicted by Clustal W for the tRNA sequences (a), and the guide tree created by neighbour-joining the scores from the pairwise alignments with gap penalty of 4 kcal/mol (b). Images produced by drawgram, from the Phylip package of programs [30].

The final method of guiding the progressive alignment, using the phylogeny predicted for the data set by Clustal W, was less successful. Comparing the final results produced from this approach versus the neighbour-joining method shows that the 'phylogeny' was a poor guide for the progressive structure alignment. For the tRNA sequences, the nearest-neighbour algorithm resulted in consensus structures with 16 or 17 basepairs for gap penalty levels ranging from 1 to 4 kcal/mol, while the structures derived from the Clustal tree had only 5 or 6 predicted pairs. The actual guide trees used at gap penalty 4 kcal/mol are contrasted in Figure 8; clearly, the order in which sequences are added to the alignment differs at a number of points.

**Figure 9:**
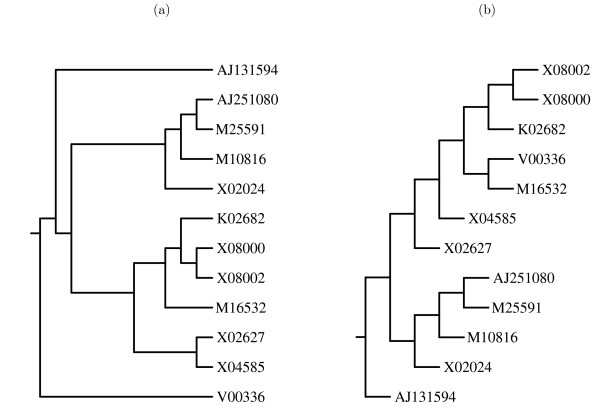
The phylogeny predicted by Clustal W for the 5S rRNA sequences (a), and the guide tree created by neighbour-joining the scores from the pairwise alignments with gap penalty of 4 kcal/mol (b). Images produced by drawgram, from the Phylip package of programs [30].

For the 5S sequences, when the Clustal tree is compared against the neighbour-joining tree based on a gap penalty of 4 kcal/mol (Figure 9), the only topological differences are that the sequence V00336 is not added until the end, and the sequences X02627 and X04585 are joined together before being aligned with any other sequences. Nonetheless, these differences were sufficient to reduce the overall consensus structure from 11 predicted basepairs to six.

The trees that were generated by the nearest-neighbour method also varied depending on the gap penalty used for the pairwise alignments; however, since we always used the same penalty for the progressive alignment as for the pairs, it cannot be specified whether changes in the final prediction are due to the guide tree or the gap penalty.

### 2.6 Using the consensus structure to constrain mfold

With such high predictive values but low sensitivity for the predicted consensus structures, the consensus structures were used as constraints for mfold, in an attempt to fill in the missing detail while still avoiding the incorrect structures that the model otherwise selected as optimal. As examples, we used the alignments created by the neighbour-joining trees at gap penalty values 4 kcal/mol and 6 kcal/mol to generate new sets of mfold predictions, with the consensus-predicted basepairs forced to be included.

**Figure 10:**
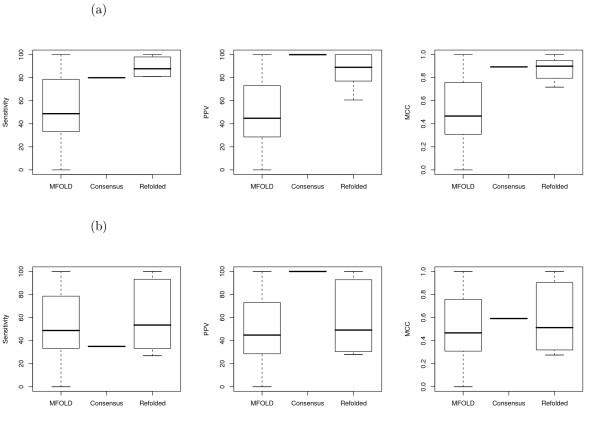
The sensitivity, positive predictive value (PPV), and Matthew's Correlation Coefficient (MCC) statistics for the unconstrained mfold optimum structure, selected consensus structures, and the mfold optimum prediction when the consensus structure is used as a forced constraint ('refolded'), for each tRNA sequence. (a) Gap penalty of 4.0 kcal/mol used to generate consensus alignment. (b) Gap penalty of 6.0 kcal/mol used to generate consensus alignment.

The consensus structure for the tRNAs that was derived for gap penalty = 4 kcal/mol had 16 predicted basepairs (compared to 20–26 basepairs per sequence in the reference structures); all the predicted basepairs were accurate, but one helix was not predicted, and another helix had been shortened by one pair. After re-folding the individual sequences with mfold, all the sequences included this missing base-pair in the optimal predicted structure, and many included the missing helix and some variable-loop helices that were not possible in the consensus structure; however, many also included incorrect additional basepairs. The change in overall sensitivity, PPV, and MCC statistics between the original mfold optimum, the consensus structure, and the constrained mfold predictions is shown in Figure 10(a). The refolded results (as measured by the Matthew's Correlation Coefficient) were also usually a considerable improvement over the worst-case results from pairwise Dynalign (for one sequence, equal to the worst case); two sequences – RS1141 and RL0503, which frequently had poor predictions from the pairwise alignments – had notable improvements over the average pairwise results.

The consensus structure calculated for the tRNA set with gap penalty 6 kcal/mol only had 7 predicted basepairs, those of the 'stem' helix in the cloverleaf structure. When the individual sequences were refolded using only these basepairs as mfold constraints, eight of the mfold optimum predictions were the same as the unconstrained predictions; this includes the sequence that was originally perfectly predicted by mfold, but also includes 3 sequences in which those seven basepairs were the only correct predictions. The constrained prediction for sequence RF6320 (which was the same as the second unconstrained prediction) also was inaccurate except for these basepairs, but this was still an improvement on the optimum unconstrained prediction, which did not contain any of the reference basepairs. Nonetheless, the constrained predictions for the other three sequences were different from any originally reported by mfold, including a perfect prediction for one (RD2640). (The changes in sensitivity, PPV, and MCC statistics for each sequence is given in Figure 10(b).)

In contrast, when the much more complete consensus structure had been used, only four of the resulting structures had been reported by the unconstrained mfold, and these mostly because of good original predictions (the worst was for RL1141, with 85% sensitivity and 61% PPV – this structure had been the second one reported by the original mfold, and both contain incorrectly predicted basepairs involving the large single-stranded variable loop region).

For the 5S rRNA data set, the consensus structure predicted by the nearest-neighbour tree was the same whether a gap penalty of 4 or 6 kcal/mol was used, so the constrained mfold predictions were only run once. This structure had 11 predicted basepairs, relative to 38–40 basepairs in the individual reference sequences (29 in the 'ideal' consensus); furthermore, one of the basepairs was incorrect for 5 of the sequences (a fact one would not usually have the luxury of knowing). Nonetheless, these constraints were sufficient to result in MCC values greater than 0.75 – 0.90 in all refolded structures except for X08000/X08002; this was an improvement over the average pairwise Dynalign results for all sequences, and it was an improvement over the unconstrained mfold in all but two cases in which it was the same prediction.

**Figure 11:**
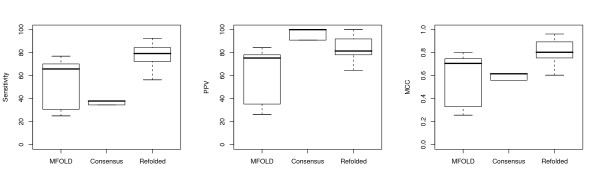
The sensitivity, positive predictive value (PPV), and Matthew's Correlation Coefficient (MCC) statistics for the unconstrained mfold optimum structure, selected consensus structure, and the mfold optimum prediction when the consensus structure is used as a forced constraint ('refolded'), for each 5S rRNA sequence.

For the (identical) sequences X08000/X08002, the refolded structure had been reported by the unconstrained mfold as a suboptimal result; with 22 correct and 12 erroneous basepairs, this was still an improvement on the original optimum, which had 12 correct and 22 erroneous basepairs. For all the other (8) sequences, the structures resulting from the constrained refolding had not originally been reported as possibilities. The statistics for the original mfold optima, the consensus structure, and the constrained optima for each sequence are in Figure 11.

**Figure 12:**
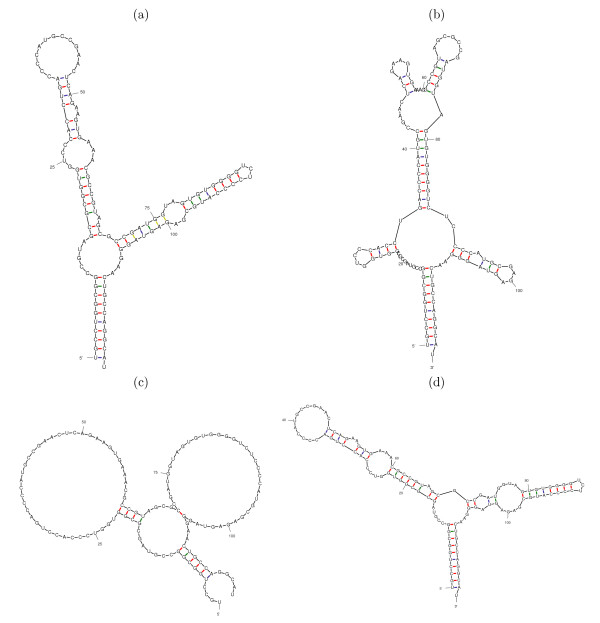
The reference secondary structure predicted for 5S rRNA sequence V00336 (a), the structure predicted by mfold as the unconstrained optimum (b), the conserved structure predicted by the nearest-neighbour consensus algorithm with gap penalty 4 or 6 kcal/mol (c), and the structure predicted by mfold as the optimum, when it was forced to include the consensus basepairs (d). Images produced by the sir graph utility of the mfold program [24].

Interestingly, the sequence with the best prediction under the constrained re-folding was also the sequence with the worst prediction, unconstrained. Sequence V00336, from *E. coli*, was originally folded by mfold into a structure in which only the outermost helix matched the reference structure (0.26 MCC); a reported sub-optimal structure likewise only matched this one helix. However, with the 11 constrained base-pairs from the consensus structure, mfold was able to detect the complete structure: all canonical basepairs were predicted, with no incorrect predictions. The reference structure, the original mfold optimum, the consensus structure, and the constrained mfold optimum are shown in Figure 12. A similar, if not quite as spectacular, improvement occurred for the sequence X02627, which went from 0.32 MCC for the original mfold prediction, to 0.90 MCC for the refold.

### 2.7 Comparison to other methods

We compared the structure prediction performance of Profile-Dynalign to other methods: the two-step Sankoff algorithm-variant PMmulti [3], and the fixed-alignment folding algorithm RNAalifold [10]. Since the quality of the input alignment can significantly effect the outcome of RNAalifold, three input alignments were considered. Alignments derived using sequence information alone were obtained using Clustalw. Then, PMmulti and Profile-Dynalign alignments were also used as input.

Table 3 shows the results ranked by average MCC score. On this particular tRNA data set, both variants of the Sankoff algorithm are making perfect predictions, however, the sensitivity of Profile-Dynalign is higher than that of PMmulti. On the 5S data set, the performance of both algorithms is comparable. The combination Clustalw and RNAalifold is performing well on both data sets, with PPV scores in the 80s. The sensitivity is lower on the tRNA set, which have lower sequence identity, and are therefore more difficult to align using sequence information alone. Consequently, the use of an alignment derived using structural information provides further improvement, particularly with respect to coverage. The combination Profile-Dynalign and RNAalifold performs best on the tRNA data set while PMmulti and RNAalifold is superior on the 5S data set.

**Table 3:** Comparing the structure prediction performance of PMmulti, RNAalifold and Profile-Dynalign on the tRNA and 5S data sets.

	**tRNA**			**5S**		
**Method**	**% Sens**	**% PPV**	**MCC**	**% Sens**	**% PPV**	**MCC**
PMmulti	30.0	100.0	0.548	36.8	88.9	0.572
Profile-Dynalign	80.0	100.0	0.894	35.9	94.7	0.583
Clustal W + RNAalifold	55.8	83.7	0.680	86.5	80.3	0.833
PMmulti + RNAalifold	55.4	88.3	0.697	96.6	85.3	0.908
Profile-Dynalign + RNAalifold	85.0	100.0	0.922	66.1	80.5	0.729

While this work was in review, a novel pairwise – stochastic context-free grammar (SCFG) approach was published [13]. In evaluating its performance, it was found that free-energy based approaches, namely Dynalign and PMcomp, are "generally best at structure prediction over the widest range" than SCFGs. Thus we expect the same trend for Profile-Dynalign.

### 2.8 Time requirements

On a 2.2 GHz processor, it required between 30 and 40 minutes to calculate a full progressive alignment for the tRNA data set (11 pairwise alignments of sequences between 71 and 92 nucleotides long, with maximum separation 25). The linear trees, in which a single sequence is added to the profile at each step, were consistently faster than the balanced trees. The calculation of a full set of pairwise alignments (66), with maximum separation 25, required over five hours; the fast pairwise alignments, with maximum separation 5, were completed in approximately ten minutes.

The 5S rRNA sequences, which were longer (117–122 nucleotides) but were aligned with a maximum separation of 15, were run on much slower 900 MHz processors; it required up to five and a half hours to calculate a full progressive alignment, although the linear trees again consistently finished faster (in less than four hours). A complete set of pairwise alignments with maximum separation of 15 required 38 hours of processor time, but with maximum separation of 5 the pairwise results were complete in just over two and a half hours.

## 3 Conclusion

We were attempting to build a reliable tool for generating secondary structure predictions for a family of RNA sequences by applying a progressive pairwise approach to the alignment and structure prediction program Dynalign. This approach, originally suggested by the creators of Dynalign when they first introduced the program [5], was inspired by past success in applying progressive pairwise alignments when creating a multiple alignment of sequences with conserved primary structure, as in the Clustal series of programs. However, the comparison is not perfect: while the primary structures being aligned by Clustal are unambiguous, the RNA secondary structures we are attempting to align are disguised in an overlapping array of potential basepairs, for which the relative likelihoods can only be estimated.

The pairwise Dynalign method on its own is in some cases sufficient to detect a true energetically stable, conserved structure between two sequences, but in other cases the optimization model still favours an alternate common potential structure. We have shown that by increasing the number of sequences used, it becomes very unlikely that the energy optimization model will predict secondary structure elements that are not part of the true conserved structure. However, it has also become clear that the progressive pairwise approach we use is strongly dependent on the order in which sequences are aligned, and that a less-than-optimal order can often result in the program not being able to detect any conserved structure at all.

When comparing our results against those from the single-sequence mfold method, the two sequence Dynalign, and the three sequence X-Dynalign, all of which are based on the same general optimization model, there are inverse trends of increasing predictive value and decreasing sensitivity as the number of sequences increases. This suggests that there might be an optimum data set size at which the combination of the two is maximized. Although this might be true for a complete dynamic programming optimization, for our progressive pairwise approach, the largest losses in sensitivity and increases in PPV tended to occur simultaneously, usually when the alignment had between 3 and 6 sequences. Although there were occasional drops in sensitivity beyond that (particularly when two six-sequence profiles were joined in a balanced tree), and some cases where PPV was still poor at six sequences (particularly in the 5S rRNA data set), the quality of prediction mostly stayed fairly steady after that point (Figures 4 and 5), suggesting that the initial alignments mostly control the final sensitivity.

Since the experiments so clearly emphasize that the tree structure used to guide the progressive alignment strongly influences the quality of the final prediction, it would be useful if we could detect the properties which differentiate a good tree from a poor one. We had examined the effect of differences in tree topology for a randomized sequence order: whether it was better to align profiles of equal numbers of sequences at each stage (balanced tree), or to add one sequence at a time to the alignment (linear tree): for the tRNA data set, there was a clear benefit to the linear approach. By examining the progression of intermediate structures (not shown), the good predictions resulted from the creation of accurate alignments early on, which were then maintained as new sequences were added. In the other cases, the progression quickly degenerated to little or no predicted consensus structure, indicating an incorrect alignment which no additional sequences could improve. When using a balanced guide tree, there would be a greater possibility that at least one of the initial profiles contains an incorrect alignment, thereby explaining the frequently poorer results.

The same split in end results was not observed for the 5S rRNA data set, for which both topologies result in low-sensitivity consensus structures, if any; this can probably be explained by the lower quality initial pairwise predictions for this data set (Figure 2 vs Figure 3).

Comparing the results of the two datasets using different algorithms (Table 3), it is clear that the Profile-Dynalign method offered the greatest improvement in predictions for the tRNA dataset. Although it is risky to derive generalisations from two examples, this is consistent with other studies [14] which suggest that structure-based alignment algorithms are the optimum choice only when sequence identity is below 60%.

The tRNA dataset had only 6 nucleotides conserved across all 12 sequences, and approximately 25% conserved across at least 8 sequences. In contrast, the 5S rRNA dataset had approximately 25% of the nucleotides conserved across all sequences, and approximately 60% conserved across at least 8; see Tables 1 and 2.

In an attempt to improve the quality of the initial alignments (and therefore the overall predictions), we also considered methods to group the sequences by similarity, so that the program aligns more closely related sequences first, merging more diverse structures once the initial alignments are established. The neighbour-joining technique, based on the pairwise alignment energy scores, seems to be a promising approach to consistently producing a reasonably good guide tree. Although this approach mimics the method in which Clustal builds its guide tree for progressive primary sequence alignment, it should be noted that while the pairwise scores in Clustal emphasize similarity in sequence, the pairwise Dynalign scores are a combination of similarity (the detail of the consensus structure) and the predicted stability of the common structures, so that highly stable structures are favoured to anchor the alignment. However, there is also the possibility that the common structure predicted by Dynalign does not reflect the true conserved structure.

Similarity in primary sequence can therefore lead to poor initial alignments, since it increases the likelihood of a common incorrect 'optimum' structure. For the tRNA data set, which had very little conserved primary sequence, this was not a problem, and the neighbour-joining technique usually resulted in a good alignment and structure prediction. However, for the 5S rRNA sequences, which had greater sequence similarity, the partial alignments from the neighbour-joining trees frequently included complex but biologically irrelevant predicted structures for alignments of up to 5 sequences (data not shown). It was only when the entire, diverse data set was aligned that the improved positive predictive value of the multiple alignment became apparent, but the incorrect early alignments then resulted in a much reduced or nonexistent consensus structure. Nonetheless, in the cases in which a structure was predicted (at gap penalties of 4 or 6 kcal/mol), it was more detailed than any of the other predictions, suggesting that the intermediate alignments, if not the structure predictions, were still better than if a random input order had been used.

One limitation of using the neighbour-joining tree based on pairwise Dynalign scores is that it can take a considerable amount of computational time to generate these scores, especially as the size of the data set increases. For a data set of *k *sequences, there are *k*(*k *- 1)/2 different pairwise alignments to compute, as compared to the *k-*1 profile alignments used in the final tree. For this reason, we also considered faster methods to approximate this structure.

The use of a phylogeny predicted by Clustal W (based solely on primary sequence) was not successful: for both data sets, the final result was no better than that for randomized trees. Applying the neighbour-joining algorithm to a set of pairwise Dynalign scores created with a time-saving reduced maximum separation parameter was a more successful option. Although these restricted pairwise alignments may not detect all the conserved structure between the most diverse sequences (for example, tRNA sequences with and without inserts in the variable loop region), they seem to be able to approximate the degree of similarity between the various sequences.

Without a definitive method with which to design a guide tree for the progressive alignment, the only way to guarantee a good prediction seems to be to experiment with different possible arrangements. That does not necessarily require an exhaustive series of complete alignments as was done here. Instead, a 'branch-and-prune' approach could be used, such that a number of the most promising pairwise alignments could be expanded into three- or four-sequence alignments, and then these increased further, at each stage dropping any partial alignments that showed a sharp decrease in the detail of the prediction. This general approach has been suggested before for RNA secondary structure detection, particularly in cases where there is a possibility of un-related sequences contaminating the data set [15].

Because the eventual predictive value of a profile structure is so high, selecting alignments by the number of basepairs and/or the estimated structural energy would probably be sufficient to guarantee optimal predictions. Mathews has also recently introduced a newer version of Dynalign which reports alternate alignments with sub-optimal energy scores [16]; if this calculation could be incorporated into the profile version of the program, it would allow for increased number of partial alignments from which to select, without nearly as much time consumed as for comparing two new profiles.

Finally, there remain a number of ways to optimize the profile alignment algorithm, particularly in the way gaps in the alignment are handled. For a primary sequence-based alignment, each position in the profile has a distinct identity. In contrast, for our structure-based alignment, large section of the profiles are aligned at once in loop regions of the structure; if these sections are of different length, gaps must be added, but it is not always clear where they should go. In our current version of the program, we have maintained the original Dynalign's default of placing the gaps at the end of the aligned regions, but this usually results in gaps directly adjacent to the start of a helix. This then causes difficulties in the next round of the progressive alignment, because the energy calculations for the stability of a helix include terms acknowledging the stabilizing effect of certain 'stacked' nucleotides adjacent to the ends of the helix. At present, when a gap appears as the adjacent 'nucleotide' in a profile, it is treated as if it was a break in the sequence, resulting in much poorer energy estimates (and very few predicted basepairs directly adjacent to existing gaps).

One solution would be to scan the sequence to find the true adjacent nucleotide, and use that in the calculations, but this would interfere with the general logic of the nearest-neighbour dynamic-programming model. Another solution is to simply avoid placing gaps next to known helices, whenever possible, by locating them in the middle of predicted loop regions.

Logically, it should also be preferable to place a gap at a position where there are already gaps in the opposing profile. This is accomplished in a primary-sequence alignment program simply by giving a neutral score to an alignment of a gap against gap. Unfortunately, this approach cannot be directly applied to the structure alignment, since the score we are optimizing is the sum of the folding energy of each sequence rather than a sum of all possible pairwise comparisons, and also because of gaps assigned to a region of the profile rather than a particular column.

A more complex solution would be to incorporate more sequence information into the alignment process, such as is used by more recent versions of the program Foldalign [17]. Not only would this help to more accurately locate gaps in the alignment, but it would also improve the alignment of unstructured (loop) regions. This could potentially improve the results of future rounds of the progressive algorithm, and possibly ameliorate the algorithm's performance on high-sequence similarity datasets.

A final issue related to gaps is the optimization of the gap penalty term. We knew, from the original Dynalign experiments and from our work on X-Dynalign, that very small or no penalty led to poorer predictions, but once an optimum was reached, additional increases in the penalty value only slightly reduced the results. We confirmed the same general trends for the profile results, except that the loss in prediction quality with too-small gap penalties became exaggerated by the progressive alignment process. We found that penalty values of between 1 and 4 kcal/mol usually gave fairly consistent results, but that higher values are preferred when the primary sequences are highly similar.

Although our method is certainly not at the point of being a consistently useful automated tool for predicting RNA secondary structure, we have found that the results it does return are highly reliable. As mentioned, it was expected from the beginning that for most data sets, even the best possible consensus structure would not include all the structural details of the individual structures. We have shown how the high predictive value of the consensus can be used to good effect in combination with the forced constraints option of mfold to create predictions with both sensitivity and reliability. However, we also discovered that if the consensus constraints do not have sufficient detail, they are less likely to improve on mfold's initial prediction, so there is still a need to increase the sensitivity of the profile structure predictions.

We have also shown how the structure-based alignments generated by this method can be used as input for the fixed-alignment covariance-based folding algorithm RNAalifold; the resulting predictions are greatly improved, relative to using a sequence-based alignment, where there was low primary-sequence identity in the data set (i.e. for the tRNA data set). However, in the case where a fairly accurate alignment could be generated directly from the primary sequence (i.e. for the 5S rRNA set), applying the RNAalifold algorithm directly to this alignment produced better structure predictions than using the Profile-Dynalign alignment.

## 4 Methods

### 4.1 Benchmark data sets

To benchmark the results of the programs, two different sets of twelve related sequences were used. The first set consists of tRNA sequences used by Mathews and Turner in their original description of the performance of the Dynalign program [5]. The second consists of bacterial 5S rRNA sequences compiled to test the three-sequence extended-Dynalign [12]. Both data sets emphasize sequences which are poorly predicted by single-sequence methods such as mfold, and both include sequences with low relative sequence identity.

The sequences and reference structures for most of the tRNAs were derived from the database maintained by Sprinzl *et al. *[18,19], except for two (RE7681 and RF6320) which were derived from the ERPIN training set [20]. The reference alignment is from the Sprinzl database, with the other two sequences added by hand. The actual tRNA molecules include a number of modified nucleotides: these have been replaced in the data set sequences by a related canonical base.

The 5S rRNA sequences and reference structures and alignment are from the Comparative RNA Web Site [21,22]. Many of the sequences contain poorly identified bases at the ends of the molecules: these have been maintained in the test sequences, and the programs will not allow them to participate in base-pairs.

The considerable time complexity of Profile-Dynalign limits its application to short sequences. Furthermore, Doshi *et al. *showed that free-energy minimization approaches using nearest-neighbor energy parameters are more reliable when the contact distance between base pairs is 100 nucleotides or less [23].

### 4.2 Baseline predictions

For comparison purposes, structural predictions for each sequence were made by the single-sequence nearest-neighbour energy model program mfold (version 3.2 [24,25]). All default parameters were accepted, including those that influence the number of sub-optimal structures reported.

In addition, predictions for all possible pairwise comparisons using the Dynalign algorithm were calculated, for seven gap penalty values: 0.0, 0.5, 1.0, 1.5, 2.0, 4.0, and 6.0 kcal/mol per inserted gap (the gap penalty values are expressed in energy units since they are being added to the empirical thermodynamic energy terms). A maximum separation of 25 was used for the tRNA data set, while a maximum of 15 was used for the 5S rRNAs; inserts within helical regions (which can be prohibited in Dynalign) were allowed. The calculations were made using the profile implementation of Dynalign that we had created (described in the next section), but the results should be the same as for the original implementation with the same parameters.

Experiments using PMcomp/PMmulti, RNAali-fold and Clustalw were run according to the author's recommended/default parameters.

### 4.3 Profile-Dynalign

Three sets of recurrence equations define the objective function: *W*, *V *and *W*5. Equations of the form *W *(*i*, *j*; *k*, *l*) represent the minimum free-energy for the optimal alignment and structure prediction of *S*_1_[*i*..*j*] and *S*_2_[*k*..*l*], when *i *and *k *are aligned, and *j *and *l *are also aligned, where *S*_1 _and *S*_2 _are the two sequence profiles being aligned and *S*_*i *_[*a*..*b*] represents the fragment comprising the the columns *a*, *a *+ 1, ..., *b *of *S*_*i*_. Equations of the form *V*(*i*, *j*; *k*, *l*) represent the minimum free-energy assuming that *i *and *j*, and *k *and *l*, are simultaneously aligned but also that *i *: *j *forms a base pair, and *k *: *l *forms a base pair. Finally, *W*5(*i*, *k*) represents the minimum free-energy for the prefix alignment of *S*_1_[1..*i*] and *S*_2_[1..*k*]. The algorithm has two steps: fill and traceback. The matrices *V *and *W *are filled by considering every 5-mer (smallest hairpin structure), 6-mer, 7-mer, and so forth up to length |*S*_1_|. Whenever *i *: *j *or *k *: *l *is a non-canonical base pair, then *V *is set to a large positive free energy value (infinity). If the two pairs *i *: *j *and *k *: *l *can form canonical base pairs (A : U, G : C, or G : U) then *V *is the minimum of three terms: the segment forms a hairpin, the segment forms a single helix or the segment forms a multi-branch loop structure. A detailed description of the different structural possibilities considered at each stage of the Dynalign algorithm, and the corresponding energy terms, is given by Mathews & Turner [5].

In order to convert the program to align profiles made from pre-calculated sequence alignments, a number of changes have been made. First, in order for a basepair interaction to be considered between two points in an profile, a canonical basepair must be possible between the nucleotides at those points for all sequences in the profile. Second, the estimated energy value for any potential structure (including an inserted gap in the alignment) is the sum of the corresponding energy values for each sequence in the profile when fit to that structure. Third, when an existing gap is encountered in one of the input alignments, it is treated equivalent to an 'intermolecular linker' character in the original program (used to model a single structure made from more than one distinct molecule).

The profiles used as both input and output in the program are in the Stockholm 1.0 alignment format (as defined in [26]), which is an annotated sequence alignment format which allows for multiple 'mark-up' lines describing the file or individual sequences, including column-by-column annotation. Gaps in the alignment are indicated by the '-' (hyphen) character.

Although the specifications for Stockholm format allow for sequences that are wrapped across multiple lines, for ease of file parsing the Profile-Dynalign program expects each sequence to be on a single line. When reading in profiles, the program also looks for a file mark-up line of type 'ID' containing an identifier with which to describe the profile as a whole in program output. When printing the final alignment to file, the program adds additional mark-up describing the parameters used by the program, the number of sequences in the alignment, the calculated energy values (both the overall optimized value and an average structural energy which is independent of the number of sequences or the gap penalty used), as well as the predicted secondary structure in bracket notation. In addition, the program also creates output files describing the predicted structure for each individual sequence in connect format. Note that the predicted structure from one round of the program does not influence the next round, except through the resulting sequence alignment.

### 4.4 Building alignment trees

A series of different scripts were created, which take as input a set of sequences (in the format described above), and arrange them into a binary tree structure to be used as a guide for the progressive application of the Profile-Dynalign program. We initially considered randomized arrangements of the input sequences, fitting them to one of two possible tree topologies: a balanced tree and a linear (degenerate) tree.

The balanced tree results in multiple alignments of a few sequences each which are then paired together to create larger alignments: at each stage, the number of sequences in each input profile is equivalent (± 1 sequence). The linear tree, in contrast, starts with an alignment of a pair of sequences, and then adds one sequence at a time to extend the alignment. Three samples of each topology were run for the data sets at each of the seven gap penalty values used for the pairwise alignments.

We also explore heuristics for predicting an optimal guide tree. As an initial approximation, we ran the Clustal W program [4] (using an interface built into the EMBOSS package of programs [27]), to predict a phylogeny for the sequences, and used this tree as a guide for the progressive structure predictions. This tree is not the same guide tree which Clustal uses to build its own progressive alignment, but rather a re-evaluated phylogeny based on the resulting profile. However, because the algorithms Clustal uses to build the alignment and to create the phylogeny both only consider the degree of primary sequence conservation, the resulting tree is only a rough estimate of the true relations between the sequences – after all, if primary sequence alignment produced reliable results for structural RNAs, there would be no reason to calculate simultaneous alignment and structure predictions.

A more thorough approach to generating an optimal tree structure was designed, using a complete set of pairwise Dynalign alignments. The approach is similar to that used to create the internal guide tree in the Clustal algorithm, in that the matrix of pairwise scores is used as the basis for a neighbour-joining tree building algorithm. The neighbour-joining tree ensures that, at each node of the final tree, the average pairwise score for the sequences in the sub-tree stemming from one branch of that node compared with the sequences in the other branch, will be better than their average score when compared against the sequences in any other subtree stemming from a node higher up in the tree. A key difference compared to the Clustal guide tree is that, for Clustal, the best score is the alignment score for the most similar primary sequences, while for our program, the best score is the estimated change in free energy of the predicted optimal consensus secondary structure.

We also created progressive alignments from neighbour-joining trees based on pairwise scores generated more quickly using the much more restrictive maximum separation value of 5 (instead of 15 or 25).

### 4.5 Constrained re-folding using mfold

For a few sample consensus structures, we used the predicted basepairs for each sequence as a set of constraints for additional mfold predictions, forcing the program to only consider potential structures which include the consensus basepairs. This technique was used previously by our lab to re-assess the results from Seed, a program to find common secondary structure motifs in a large data set [28].

### 4.6 Implementation and availability

All the experiments for the tRNA data set were executed on 2.2 GHz AMD Opteron 248 processors with a Solaris 9 operating system and approximately 4 Gb RAM per processor. The 5S rRNA sequences were run on 900 MHz UltraSPARC III processors, also with a Solaris 9 OS and at least 4 Gb RAM per processor.

The Profile-Dynalign program is implemented as a shell-based C++ program, and was compiled with the native Solaris C++ compilers on the above-mentioned systems. The routines to create tree structures and run the alignments in a progressive fashion were implemented as Perl scripts. The source code as well as the scripts are made available under the GNU General Public Licence [29].

## Authors' contributions

ABBR made several contributions to Profile-Dynalign source code, designed and implemented the approaches for building the guide trees, ran the tests and wrote the initial draft of the manuscript under the supervision of MT. Both authors have read and approved the final manuscript.
